# Genome-Wide Identification and Expression Analysis of Fatty Acid Desaturase (*FAD*) Genes in *Camelina sativa* (L.) Crantz

**DOI:** 10.3390/ijms232314550

**Published:** 2022-11-22

**Authors:** Daqian Sun, Weizhu Quan, Di Wang, Jingyan Cui, Tianyi Wang, Mei Lin, Yijin Wang, Nan Wang, Yuanyuan Dong, Xiaowei Li, Weican Liu, Fawei Wang

**Affiliations:** College of Life Sciences, Jilin Agricultural University, Changchun 130118, China

**Keywords:** *Camelina sativa* (L.) Crantz, fatty acid desaturase (FAD2-2), gene family, low temperature (4 °C), linolenic acid (C18:3)

## Abstract

*Camelina sativa* (L.) Crantz is an indispensable oilseed crop, and its seeds contain many unsaturated fatty acids. FAD (fatty acid desaturase) regulates the synthesis of unsaturated fatty acids. In this research, we performed *CsFAD* gene family analysis and identified 24 *CsFAD* genes in Camelina, which were unevenly distributed on 14 of the 19 total chromosomes. Phylogenetic analysis showed that *CsFAD* includes four subfamilies, supported by the conserved structures and motifs of *CsFAD* genes. In addition, we investigated the expression patterns of the *FAD* family in the different tissues of Camelina. We found that *CsFAD* family genes were all expressed in the stem, and *CsFAD2-2* was highly expressed in the early stage of seed development. Moreover, during low temperature (4 °C) stress, we identified that the expression level of *CsFAD2-2* significantly changed. By observing the transient expression of CsFAD2-2 in Arabidopsis protoplasts, we found that CsFAD2-2 was located on the nucleus. Through the detection and analysis of fatty acids, we prove that CsFAD2-2 is involved in the synthesis of linolenic acid (C18:3). In conclusion, we identified *CsFAD2-2* through the phylogenetic analysis of the *CsFAD* gene family and further determined the fatty acid content to find that *CsFAD2-2* is involved in fatty acid synthesis in Camelina.

## 1. Introduction

*Camelina sativa* (L.) Crantz (Camelina), which is an indispensable oilseed crop in the northern regions of the earth, can survive normally at −2 °C [1,2]. In recent years, many studies have shown that Camelina is more suitable for planting in autumn to increase the oil content of the seeds [2,3]. Meanwhile, the oil content of Camelina seeds accounts for 35% of dry weight [4]. Camelina has received more attention because of its good agronomic traits and can provide a large number of high-quality fatty acids [3,5,6,7,8]. A growing number of studies have shown that the ω-3 fatty acids, which are an important component of the cytoplasmic membrane, such as hexadecatrienoic acid (C16:3) and α-linolenic acid (C18:3), will accumulate in plants when exposed to low temperature [9,10,11,12]. The content of α-linolenic acid in Camelina seeds is close to 40%, which can improve the tolerance of these plants to abiotic stress [13,14]. In conclusion, the content of fatty acid in Camelina is relatively high compared with other species, especially the content of linolenic acid, which accounts for a relatively large proportion of the total fatty acids contained in Camelina seeds.

The intracellular pathway of fatty acid desaturation is generally regulated by FADs (fatty acid desaturases) in plants, including Camelina [9,12,15,16,17,18]. Desaturase activity in fatty acid biosynthesis was originally discovered in the cytoplasm of the different tissues of the plant [19]. Although FAD activity in the cytoplasm has been demonstrated, these enzymes are located in different cell compartments [20]. In Arabidopsis, FAD2 and FAD3 are localized to the ER (endoplasmic reticulum), whereas FAD4, FAD7, and FAD8 are only found in the plastid [20,21,22,23,24]. It has been shown that FAD2 and FAD4 may be associated with fatty acid synthesis or degradation, and FAD4, FAD7, and FAD8 may be involved in fatty acid metabolism and transport.

Moreover, researchers also found that the protein expression of FAD3 in wheat root tips was significantly upregulated [9], and the accumulation of ω-3 fatty acids (C18:3) was also achieved through the upregulation of FAD3 expression in low temperatures. In addition, the acetylation of the histones of FAD3 also affects fatty acid biosynthesis in seeds [25]. FAD5 is required for hexadecenoic acid (C16:3) synthesis in the chloroplasts of *Marchantia polymorpha* [15]. Notably, FAD5 regulates autoimmunity in plant cells by catalyzing palmitate acid [12]. The chloroplast-localized FAD8 was more selective for C18:2 and the function of catalyzing the production of C18:3 was non-redundant with FAD7 at 22 °C [20]. Furthermore, previous studies have demonstrated that FAD3 and FAD7 are involved in lipid transport between the ER and chloroplast and also revealed the differential contribution of FADs with different localization to linolenic acid [26]. Increasing the expression of *FAD3* and *FAD7* increases the C18:3 accumulation in the tomato, which will further promote plants to have stronger resistance to cold stress [27]. In terms of plant response to low-temperature stress, both *fad2* and *fad6* mutants showed growth defects, and the accumulation of C16:3 and C18:3 was significantly reduced in vivo [28,29,30]. In plants, the double bonds in the vast majority of fatty acids are associated with membrane lipids with the *cis* configuration. However, the particularity of FAD4 is that the double bonds of the catalyzed fatty acid have the trans configuration [24]. Meanwhile, the C16:1 catalyzed by FAD4 will participate in the redox reaction of chloroplasts [17]. In summary, most studies have established that *FADs* are associated with fatty acid metabolism, especially since they catalyze the production of unsaturated fatty acids. 

In the *FAD* gene family, *FAD2* has relatively undergone little research. In Arabidopsis, FAD2 catalyzes oleic acid (C18:1) to linolenic acid (C18:2) [31]. FAD2 regulates Arabidopsis response to the endoplasmic reticulum stress by controlling linolenic acid accumulation [16]. The DsFAD2 (Dimorphotheca sinuate) enzyme has a high preference for linolenic acid, indicating that it is closely related to the synthesis of linolenic acid [32]. Linolenic acid (C18:3) can improve plant resistance to low-temperature stress. Moreover, *fad2* mutants cannot grow normally at low temperatures [28]. We sought to investigate whether FAD2 could regulate fatty acid synthesis in Camelina. Here, we identified and analyzed the *FAD* gene family of Camelina at different developmental stages and performed further quantitative analysis of the *FAD* gene family under low-temperature stress. The analysis of the gene family includes phylogenetic analysis, gene structure analysis, and the analysis of chromosomal locations. Moreover, we also determined that *CsFAD2* can improve the resistance of Camelina to low-temperature stress. Meanwhile, subcellular localization was investigated to determine the expression of CsFAD2. Furthermore, we used Pichia pastoris as a receptor for the heterologous expression of *CsFAD2* to detect the changes in fatty acid content. We found that *CsFAD2-2* can regulate fatty acid synthesis in plants. Meanwhile, we analyzed the expression patterns of the *FAD* family and found that *FAD2-2* has a certain relationship with low-temperature stress.

## 2. Results

### 2.1. Genome-Wide Identification and Analysis of the CsFAD Gene Family

Camelina is an allohexaploid plant. All the gene sequence information of the *CsFAD* family was found in the NCBI database. The sequence analysis of the *FAD* gene family was carried out using bioinformatic methods. According to the different relationships, the genes of the 24 *CsFAD* families that may be related to the fatty acid synthesis of Camelina seeds were divided into seven categories, namely *CsFAD2*, *CsFAD3*, *CsFAD4*, *CsFAD6*, *CsFAD7*, *CsFAD8*, and *CsSLD*, respectively. The shortest gene length is the *CsFAD4-5*, and the longest is the *CsFAD 7-1*. Subsequently, the amino acid sequence analysis of the *CsFAD* family showed that the sequence was 408-613 amino acids in full length, and the molecular mass was 38.7–129.8 kDa (Table 1).

### 2.2. Phylogenetic Distribution of FAD Genes in Camelina, Soybean, and Arabidopsis

**Figure 1:**
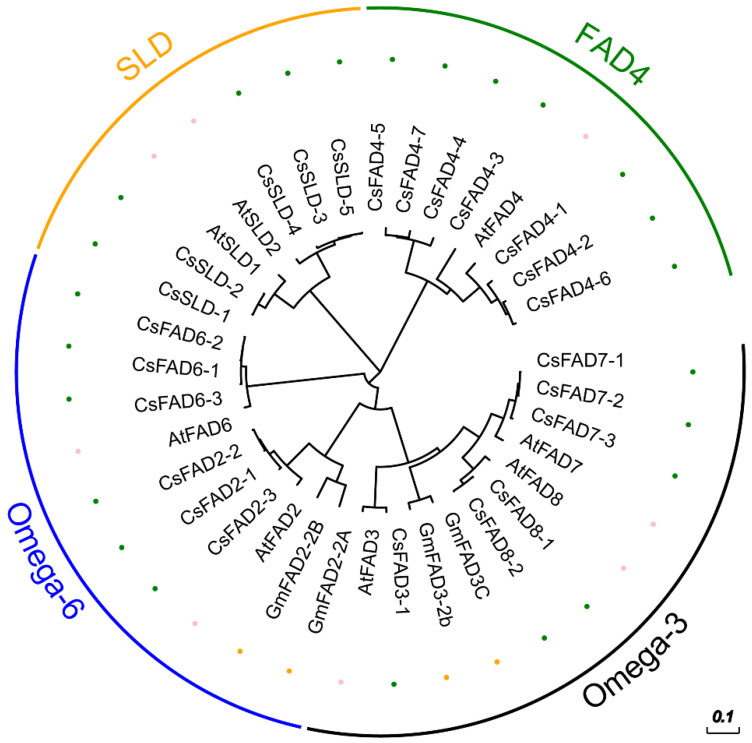
Phylogenetic analysis of FAD proteins in Camelina sativa, Arabidopsis thaliana, and Glycine max. Neighbor-joining phylogeny of 36 FAD genes in the 3 species was analyzed by using MEGA 7.0 with 2000 times bootstrap replications. Tree scale is 0.1. FAD family was divided into four subfamilies, namely Omega-3, Omega-6, FAD4, and SLD, which are labeled in the outermost layer. Three different colors were selected to represent three species.

To compare the phylogenetic distribution of *FAD* genes, which is related to fatty acid dehydrogenase in Camelina, soybean, and Arabidopsis, we constructed a phylogenetic tree using MEGA7 (Figure 1). CsFAD2-2 was found to be closely related to CsFAD2-1 and CsFAD2-3 and had different branches and relatives of Arabidopsis AtFAD2, soybean GmFAD2-2B, GmFAD2-2A, and other genera. However, the FAD genes of Camelina were divided into four subfamilies. The *DES1* subfamily genes were not identified in Camelina. Therefore, the DES1 genes may have been lost in Camelina during evolution. *Omega-3* was the largest of the four subfamilies. *FAD4* was the most special subfamily, and no *FAD4* was found in soybean [33] (Figure 1, Table S7), suggesting that *FAD4* is likely lost in soybean. Furthermore, most of the soybean *FAD4* tended to be amplified after the ancestral genes diverged. By contrast, Camelina contained high amounts of *FAD4*.

### 2.3. Gene Structure Analysis and Chromosomal Location of FAD Gene Family in Camelina

**Figure 2:**
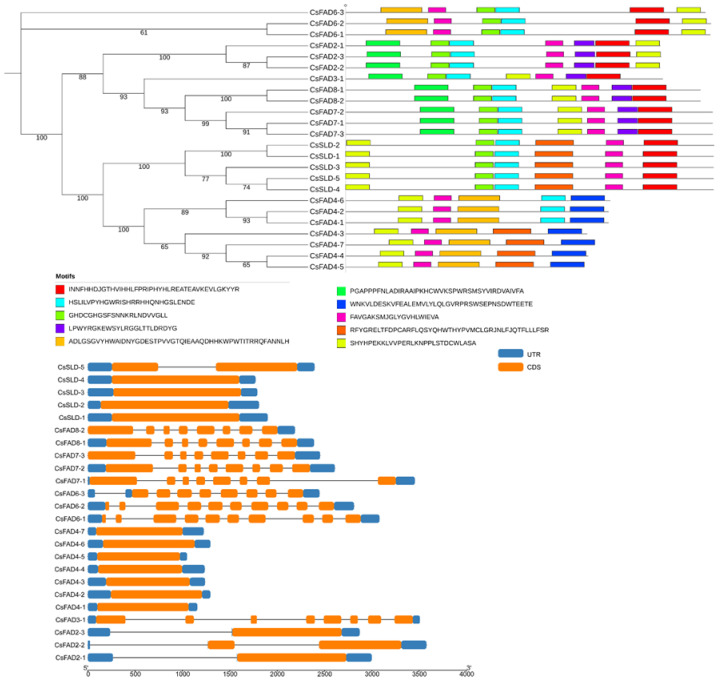
The motifs and structures of FAD *gene* family from *Camelina sativa*. MEME was used to analyze the protein sequence, and 10 motifs were predicted in FAD sequence. Conservative domains are represented by different colors, and the black lines are non-conservative domains. Scale bars represent 50 amino acids. Yellow represents CD sequences, black lines represent introns, and blue represents non-coding regions (UTR). Scale bars represent 500 bp.

To characterize the sequence structure of the *CsFAD* gene family, the coding sequence of the *CsFAD* gene was analyzed, the amino acid sequence of the *CsFAD* gene family was used to generate a separate phylogenetic tree, and the exon and intron structures of these genes were compared (Figure 2, Table S4). The results showed that except for CsFAD2-1, CsFAD4 and CsSLD1, CsSLD2, CsSLD3, and CsSLD4, the rest of the genes contained different numbers of introns, among which CsFAD2-2 and CsSLD5 each had one intron. Meanwhile, CsFAD3, CsFAD7, and CsFAD8 each had seven introns, and their conserved genetic structure supported their close evolutionary relationship. CsFAD6-3 had eight introns, while CsFAD6-1 and CsFAD6-2 had nine introns each (Figure 2). The results of protein functional domain analysis showed that, in addition to CsFAD4, the genes that are more closely related are more similar in their protein functional domain structure. It can be seen from the structure of exon–intron that CsFAD2-2 is obviously different from CsFAD2-1 and CsFAD2-3, which may indicate that CsFAD2-2 performs different functions from CsFAD2-1 and CsFAD2-3. 

**Figure 3:**
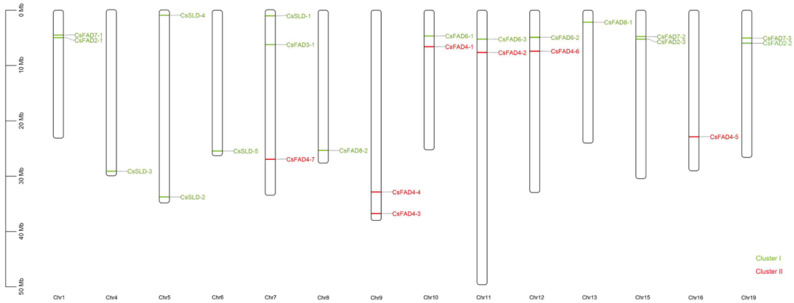
TBtools was used to draw the maps of chromosome location. Uneven distribution of *CsFAD* genes on 14 chromosomes. Cluster I represent fatty acid desaturase (FAD) that can catalyze *cis*-double-bond fatty acids. Cluster II represents fatty acid desaturase (FAD) that can catalyze *trans*-double-bond fatty acids.

In addition to the exon–intron analysis, conserved motifs may also be important for the various functions of the *CsFAD* genes. Using TBtools, we analyzed and determined that *CsFAD* had nine conservative motifs. The length of the motif ranged from 21 to 50 amino acids, and the number of CsFAD motifs ranged from 4 to 6. CsFAD2-1, CsFAD2-2, and CsFAD2-3 all contained six conservative motifs. Overall, the Camelina CsFAD family contained eight motifs (Figure 2). The protein motifs and protein functional domain analysis of the *CsFAD* gene family showed that the more closely related the genes were, the more similar the protein functional domain structure would be. The *CsFAD* genes were distributed on 14 chromosomes (Figure 3), and *CsFAD4-7*, *CsFAD3-1*, and *CsSLD1* were located on chromosome 7. Chromosomes 1, 5, 9, 10, 11, 12, 15, and 19 contained a significant number of *CsFAD* genes. According to the results of the gene location on chromosomes, it was revealed that CsSLD and CsFAD4 were randomly distributed on different chromosomes, but on chromosomes 10, 11, and 12, CsFAD4 and CsFAD6 existed at the same time, indicating that there may be some connection between these two subfamilies. At the same time, CsFAD2 and CsFAD7 may also have such a connection, but the specific association analysis needs to be further proved. Meanwhile, using the structural analysis results of the conservative structural domain (Figure S3), we found that CsFAD2 only contained the functional domain of Omega 6 fatty acid dehydrogenase, indicating that CsFAD2 is mainly involved in fatty acid metabolism. CsFAD3, CsFAD6, CsFAD7, and CsFAD8 all contained only the membrane FADS family or superfamily functional domains, which may be involved in the synthesis of membrane fatty acids, while CsSLD contained the cytochrome b5 functional domain, indicating that CsSLD may also be related to the metabolism of cytochrome b5. CsFAD4 is different from the domain of other CsFADs because CsFAD4 catalyzes trans-unsaturated double bonds.

### 2.4. Tissue- and Organ-Specific FAD Gene Expression in Camelina 

**Figure 4:**
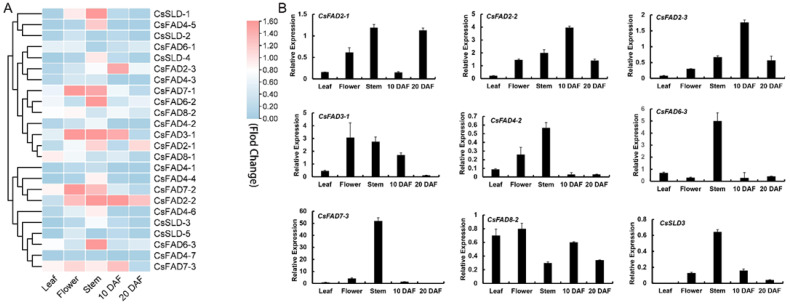
Expression profiles of CsFAD family genes in different Camelina tissues and organs: (**A**) the normalized expression levels of the hierarchical clustering of 24 CsFAD genes in 5 tissues (leaves, stem, flower, seeds 10 days after flowering (DAF), and seeds 20 days after flowering (DAF)). The transcription level was graded color scale from green to red. Expression data were obtained from the Camelina genome database. The normalized expression level of CsFAD genes is expressed in the form of log2 values; (**B**) qRT-PCR profiles of CsFAD genes in different Camelina tissues and organs. The expression level of the CsActin gene in Camelina was normalized as “1”. Vertical bars indicated the standard error of the mean. Data are shown as means ± SD of three replicates (n = 3 × 3).

We analyzed the expression levels of the *CsFAD* gene families of Camelina leaves, stems, flowers, and seeds at different developmental stages after flowering (Figure 4, Figure S1, Table S5). The results showed that the expression of *CsFAD* gene families in Camelina leaves was generally low, and there were different degrees of expression in the stem, among which the relative expression of *CsFAD2* genes in seeds was high, especially *CsFAD2-2* in seeds 10 days after flowering. It reached 2.4–100 times the expression of genes in other families. This stage of plant development is an important period for the synthesis of fatty acids in Camelina seeds, so based on this, we can reasonably speculate that the *CsFAD2-2* gene plays an important role in the fatty acid synthesis process of Camelina.

### 2.5. Expression Analysis of the CsFAD Gene Family under Low-Temperature Stress

**Figure 5:**
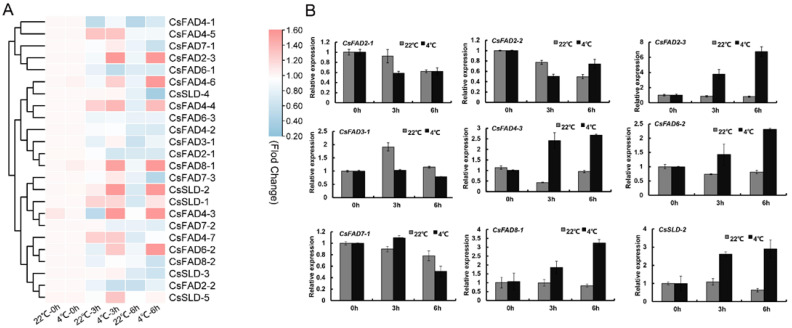
Expression patterns of CsFAD genes in Camelina during low-temperature stress: (**A**) normalized expression levels of CsFAD genes in Camelina during low-temperature stress. qRT-PCR was used to investigate the expression levels of the CsFAD genes during low-temperature stress. Log2 fold change for low-temperature treatment was used to present expression change under low-temperature treatment. Expression levels are illustrated by graded color scale from green to red; (**B**) qRT-PCR profiles of CsFAD genes during low-temperature treatment. The low-temperature (4 °C) treatment lasted 0 h, 3 h, and 6 h. The expression level of CsFADs in control was normalized as “1”. Vertical bars indicate the standard error of the mean. Data are shown as means ± SD of three replicates (n = 3 × 3).

The expression of key genes in plant fatty acid anabolism not only controls the synthesis of lipid components but is also closely related to the cold tolerance of plants. The Camelina seedlings were treated at a low temperature of 4 °C, and then the gene expression of Camelina seedlings grown at 22 °C and 4 °C was analyzed, and the expression of 15 genes in the CsFAD gene family was found to decrease to varying degrees, among which the CsFAD2 gene significantly changed during treatment for 3 h, and the expression decreased by 34.6–41.3%. It was also observed that the expression of 11 genes was increased after cryogenic treatment (Figure 5, Figure S2, Table S6). The expression of the FAD gene family was significantly upregulated in the early or late stage of low-temperature stress treatment. After 6 h of low-temperature treatment, the expression of CsFAD2-2 was significantly upregulated. The above results can prove that the CsFAD genes are related to the cold resistance of Camelina, but how these genes specifically affect the cold tolerance of Camelina remains to be investigated. The expression level of CsFAD2-2 in Camelina seeds was very high, and the CsFAD2-2 expression level increased with the increase in low-temperature treatment time. 

### 2.6. CsFAD2-2 Subcellular Localization Analysis

**Figure 6:**
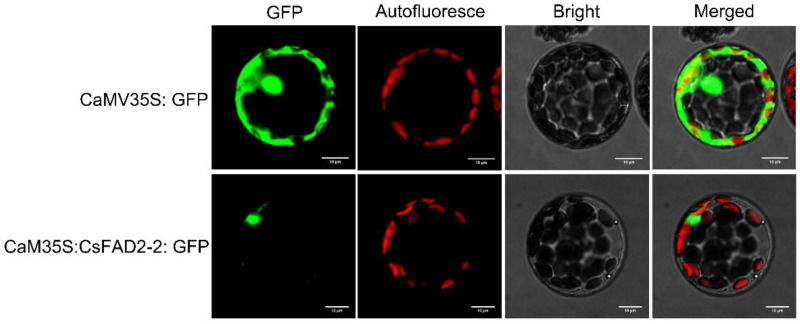
Subcellular localization of CsFAD2-2. Subcellular localization of CsFAD2-2 in Arabidopsis protoplasts. CsFAD2-2 was fused with CaMV35S: GFP to obtain the GFP–CsFAD2-2 fusion protein. The upper row shows the fluorescent signal from transforming CaMV35S: GFP vector. The bottom row shows the fluorescent signal from transforming CaMV35S: CsFAD2-2–GFP vector. Scale bars, 2 μm.

The constructed plant fusion expression vector pCAMBIA1302-CsFAD2-2 was transformed into Arabidopsis protoplasts and tobacco, the empty vector pCAMBIA1302 was transformed as a control to observe the distribution of green fluorescence signal under laser confocal microscopy (Figure 6, Figure S5). The subcellular localization results showed that all the GFP signals were observable in Arabidopsis protoplast cells in the control group of the transformed empty vector pCAMBIA1302. In the experimental group of recombinant vector pCAMBIA1302-CsFAD2-2, a strong green fluorescence signal was observed only on the nucleus of Arabidopsis protoplasts (Figure 6). It was found that the protein encoded by the *CsFAD2-2* gene was mainly distributed in the nucleus.

### 2.7. Analysis of Fatty Acid in Transgenic Yeast

**Figure 7:**
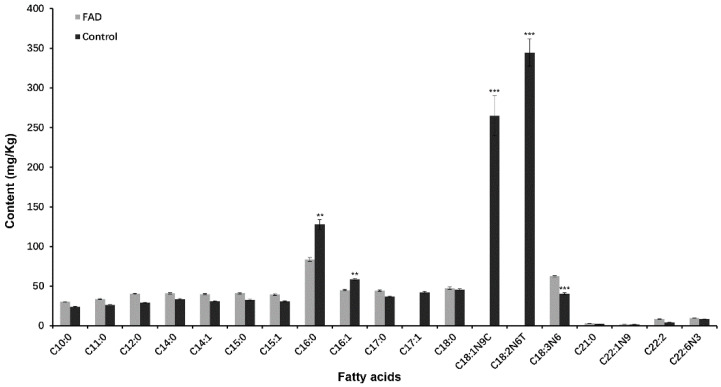
Determination of fatty acid in *Pichia pastoris*: Determination of fatty acid content in *Pichia pastoris*. Data are shown as means ± SD of three replicates (n = 3 × 3); Statistical significance analysis was carried out by performing Student’s *t*-test ( ** *p* < 0.01 and *** *p* < 0.001).

*Pichia pastoris* has a simple fatty acid structure that lacks an endogenous FAD2 activity that can be used to verify the function of CsFAD2 fatty acid dehydrogenase [34,35,36]. In *Pichia pastoris* expressing *CsFAD2-2,* gas chromatography–mass spectrometry was used to compare the accumulation of fatty acids with that in control. Different degrees of fatty acid changes were observed in recombinant yeast cells transformed with the cDNA of *CsFAD2-2* (Figure 7, Figure S4). The content of decanoic acid (C10:0) of saturated fatty acid was 29.82 mg/kg, undecanoic acid (C11:0) was 32.91 mg/kg, lauric acid (C12:0) was 39.44 mg/kg, and myristic acid (C14:0) was 39.92 mg/kg. The contents of pentadecanoic acid (C15:0) and heptadecanoic acid (C17:0) were 39.98 mg/kg and 43.2 mg/kg, respectively. However, in the control group, the content of C10: 0 was 23.77 mg/kg, an increase of 25.45%; C11: 0 was 26.39 mg/kg, an increase of 24.7%; C12: 0 was 29.44 mg/kg, an increase of 33.97%; C14: 0 was 33.49 mg/kg, an increase of 19.20%; C15: 0 and C17: 0 were 33.14 mg/kg and 36.93 mg/kg, increases of 20.64% and 16.98%, respectively, but the content of stearic acid (C18: 0) did not change significantly.

The content of myristic acid (C14:1), an unsaturated fatty acid, in recombinant yeast cells was 38.81 mg/kg, the content of C14:1 in empty yeast cells was 31.15 mg/kg, an increase of 24.59%, and the content of *cis*-10-pentanoic acid (C15:1) was 38.13 mg/kg. Although the content of *cis*-13, 16-docosadienoic acid (C22:2), *cis*-4, 7, 10, 13, 16, 19-docosahexaenoic acid (C22:6), and erucic acid (C22:1) accumulated more in transgenic yeast cells than in controls, the overall level was still low. It is worth noting that the content of linolenic acid (C18:3) increased by 47.7%, from 41.72 mg/kg to 61.6 mg/kg, and the content of oleic acid (C18:1) decreased from 294 mg/kg in the control group to 35.97 mg/kg in recombinant yeast cells, with a decrease of 87.8%. This indicates that the main function of the *CsFAD2-2* gene is to increase the unsaturated degree of fatty acids and further catalyze the dehydrogenation of oleic acid (C18:1) to linolenic acid (C18:3).

## 3. Discussion

In plants, fatty acids are a major factor in determining the value of vegetable oils. Thus, optimizing the content of high-quality fatty acids in seeds is one of the important tasks in oil crop breeding. Although there are fatty acids with different carbon chains in natural plants, most commercial vegetable oils are abundant in C16 and C18 and contain up to three unsaturated double bonds [37]. The fatty acids’ desaturating enzymes encoded by the *FAD* genes in plants catalyze and generate unsaturated fatty acids containing 2–3 double bonds, such as hexadecatrienoic acid (C16:3) and α-linolenic acid (C18:3) [38]. Recently, some studies have demonstrated that *FAD* genes can increase the oil content of oil crops [39,40]. *FAD* genes not only promote the synthesis of unsaturated fatty acids in seeds but also play a crucial role in plant resistance to abiotic stress, especially low-temperature stress [41]. There are few studies on the response of *FAD* genes to low-temperature stress, among which *AtFAD7* and *AtFAD8* have been shown to respond to low temperatures [20,27]. In Arabidopsis and cotton, *FAD2* has been shown to promote fatty acid accumulation and improve seedling tolerance to cold stress [42,43]. Nevertheless, there are relatively few studies on the function of *FAD2* in Camelina, especially its resistance to low temperatures.

In this research, we identified and compared 24 *FAD* family members in Camelina. The phylogenetic tree results showed that CsFAD2 was relatively close to AtFAD2 (Figure 1). This suggests that they may have similar evolutionary events and have approximately the same functions. It is worth noting that, compared with *Arabidopsis thaliana* and *Glycine max*, the specific FAD4 subfamily in *Camelina sativa* may be related to fatty acid synthesis, but clear experimental evidence is required to prove its correlation. Gene structure analysis showed that the structures of introns and exons of the *FAD* gene family were relatively conserved (Figure 2). The results of chromosome mapping showed that the members of the Camelina *FAD* gene family were distributed on different chromosomes (Figure 3). This indicates that *FADs* are involved in the regulation of many plant functions. 

We analyzed the expression patterns of the *FAD* gene family in the different tissues of Camelina (Figure 4). The *CsFAD4* gene had the highest expression level in the stem, and FAD4 was found to be the only fatty acid desaturase in the *FAD* gene family that catalyzes the trans double bond [17], indicating that trans fatty acid in the sporophyte may be mainly distributed in the stem or transported through the stem. Both *CsFAD7* and *CsFAD8* were expressed in flowers and stems, and in previous studies, FAD7/8 was found to promote plant resistance to low temperatures [20,22]. Therefore, we speculate that, under low-temperature conditions, *CsFAD7/8* may regulate the flowering and development of plants. The expression of *CsSLDs* was relatively high in the stems and seeds of 10 DAF, indicating that *CsSLDs* may be involved in the desaturation of fatty acids in the early stage of seed formation. Although *CsFAD2* was expressed in stems, its expression level changed most obviously in seeds; *CsFAD2-2*, in particular, was highly expressed in the early formation of seeds. The seeds synthesized unsaturated fatty acids during the formation process, indicating that *CsFAD2* may be involved in the synthesis of plant unsaturated fatty acids. Subsequently, using transgenic yeast, we verified this conjecture that *CsFAD2-2* promotes the accumulation of linolenic acid (C18:3). Meanwhile, the Camelina *FAD* family genes were found to respond to low temperatures (Figure 5). 

The expressions of *CsFAD2*, *CsFAD7,* and *CsFAD8* were downregulated at 4 °C for 3 h (Figure 5) but were significantly upregulated at 4 °C for 6 h. In contrast, the *CsSLD* was upregulated at 4 °C for 3 h. Such results suggest that the response of *CsFAD2* to low temperatures may be after *CsSLD*; in other words, *CsFAD2* is the operative gene in a low-temperature environment in Camelina.

Interestingly, AtFAD2 was located on the endoplasmic reticulum, but CsFAD2 was expressed in the nucleus (Figure 6). The reason may be that AtFAD2 is located on chromosome 1, but CsFAD2-2 is located on chromosome 19 and is located close to AtFAD7. Moreover, AtFAD7 is localized on the plastid [22], which also provides good evidence for the subcellular localization of CsFAD2-2. In this study, the fatty acid contents of transgenic CsFAD2-2 yeast cells and control yeast cells were compared and analyzed. The results showed that the contents of various fatty acids in transgenic yeast changed to varying degrees; for instance, the content of oleic acid decreased by 87.8%, but the content of linolenic acid increased by 47.7%, which proves that the main function of CsFAD2-2 is to catalyze the dehydrogenation of oleic acid to linolenic acid. Notably, in the course of the fatty acid analysis of the CsFAD2-2 group, we found that linoleic acid (C18:2) almost disappeared, in addition to the substantial reduction in oleic acid (C18:1) content. This result fully demonstrates that CsFAD2-2 can catalyze C18:2 to C18:3. In summary, CsFAD2-2 can catalyze linoleic acid (C18:2) to C18:3, thereby enhancing the resistance of Camelina to low temperatures.

## 4. Materials and Methods

### 4.1. Identification and Analysis of FAD Gene Family

The genome and protein sequences of FAD in soybean, Arabidopsis, and Camelina were downloaded from the NCBI (https://www.ncbi.nlm.nih.gov/) and Phytozome (https://phytozome-next.jgi.doe.gov/) [44] databases. The HMM profiles of the FAD domain (PF00487) were downloaded from the Pfam protein database (https://pfam.xfam.org/) for soybean, Arabidopsis, and Camelina [45]. The FAD protein sequences were submitted to the SMART database (http://smart.embl.de/) [46] for further domain analysis. Molecular Evolutionary Genetics Analysis (MEGA7) was used for multiple sequence alignment of FAD proteins in the three species [47]. The structural diversity values of intron and exon organizations were obtained from genome annotation [11]. The conserved domains and motifs of these proteins were annotated using the MEME (https://meme-suite.org/) database [48]. The final structure diagram was performed using TBtools (v1.0692) [49]. The syntenic analysis of the *CsFAD* gene family was performed using MCScanX [50]. Subsequently, TBtools was used to draw a schematic diagram of the chromosomal location distribution of the *CsFAD* gene family.

### 4.2. Plant Materials

The growth and treatment of plants were analyzed following [51], with slight modifications. *Camelina sativa* (L.) Crantz was grown at 22 °C, 50% relative humidity, and a photoperiod of 16 h light/8 h dark. The plants were regularly watered with a 1/2 Hoagland solution. The 2-week-old seedlings were treated with cold stress (4 °C). The seedling leaves of the control (22 °C) and treatment (4 °C) were taken out. Subsequently, they were frozen with liquid nitrogen (N2) and then stored at −80 °C. For the identification of the expression levels of the *FAD* gene family in different tissues, the samples included leaves, flowers, stems, seeds after 10 days of flowering (DAF), and seeds after 20 days of flowering (DAF); these samples were put into N2 for freezing treatment and then transferred to −80 °C for storage. 

### 4.3. RNA Extraction and qRT-PCR Analysis

The extraction and purification of the total RNA following the product instructions of a TaKaRa MiniBEST Plant RNA Extraction Kit (TaKaRa). The detection concentration of RNA was expressed as ng/µL. The reverse transcription of RNA followed the manual of a PrimeScript™ RT Reagent Kit (TaKaRa). TB Green Premix Ex Taq II (Tli RNaseH Plus) (TaKaRa) was used for the qRT-PCR reaction. The standard procedure for two-step PCR amplification was first 95 °C for 30 s, then 40 cycles of 95 °C for 5 s, and 60 °C for 30 s [11]. The primers required for qRT-PCR are summarized in Supplementary Table S1.

### 4.4. Subcellular Localization Assay

To demonstrate the intracellular organelle-specific presence of CsFAD2-2, *CsFAD2-2* was inserted into the pCAMBIA1302 vector using the polyclonal site *Eco*R I/*Kpn* I. The protoplasts were extracted from Arabidopsis leaves as previously described [52]. Additionally, the transient expression of CsFAD2-2–GFP fusion protein was analyzed in Arabidopsis mesophyll protoplasts. Fluorescence signals were monitored using a Leica TCS SP8 laser scanning confocal microscope (Germany). The primers required for the subcellular localization assay are summarized in Supplementary Table S2.

### 4.5. Determination of Fatty Acid (FA) Content

To determine the FA content in yeast (*Pichia pastoris*), the full-length *CsFAD2-2* coding sequence was cloned into the pPICZalphaA vector with the polyclonal site *Eco*R I/*Kpn* I. The recombinant vector was transformed into *Pichia pastoris* X33. A YPD medium containing Zeocin was used to grow transgenic yeasts. The transgenic yeasts were grown at 22 °C, 250 rpm for 3 d. The extraction of the total lipids from yeast cells followed the method of [53], with minor modifications. Subsequently, the samples were developed in methanol: CH_2_Cl_2_ (v1: v2 = 1:3) for 10 min at 30 °C. 6% The KOH in the methanol solution was added into the reaction system for 12 h. Finally, hexane: diethylether (v1: v2 = 80:20) was added to the reaction system. The extracted FA methyl ester was dried under nitrogen gas (N2). The analysis of FA methyl ester was performed according to the previous studies of gas chromatography–mass spectrometry methods [54]. The primers required for pPICZalphaA–CsFAD2-2 are summarized in Supplementary Table S3.

## 5. Conclusions

We performed a phylogenetic analysis of the CsFAD gene family and identified the expression patterns of the CsFAD gene family under low-temperature (4 °C) stress. Meanwhile, we identified CsFAD2-2 to be localized in the nucleus and found that CsFAD2-2 can catalyze the linolenic acid (C18:3) biosynthesis.

### Statistical Analysis

All data were statistically analyzed by performing Student’s *t*-test (* *p* < 0.05, ** *p* < 0.01, and *** *p* < 0.001). The data are expressed as mean ± standard deviation (SD). The physiological phenotypic data were also compared using analysis of variance (ANOVA). 

## Figures and Tables

**Table 1:** Sequence analysis of *CsFAD* gene family.

Gene ID	Sequence Number	Chromosome	Gene Location
*CsFAD2-1*	LOC104776214	1	4949,343–4952,341(-)
*CsFAD2-2*	LOC104764975	19	5522,583–5524,894(-)
*CsFAD2-3*	LOC104745425	15	5229,237–5232,158(-)
*CsFAD3-1*	LOC104700502	7	6302,189–6305,695(+)
*CsFAD4-1*	LOC104717530	10	6575,131–6577,244(-)
*CsFAD4-2*	LOC104722223	11	7634,471–7635,739(-)
*CsFAD4-3*	LOC104714552	9	36744,852–36746,085(+)
*CsFAD4-4*	LOC104713733	9	32860,862–32862,097(-)
*CsFAD4-5*	LOC104751928	16	22866,693–22867,739(+)
*CsFAD4-6*	LOC104730684	12	7388,093–7389,357(+)
*CsFAD4-7*	LOC104702825	7	27024,843–27026,085(+)
*CsFAD6-1*	LOC104717096	10	4649,200–4652,284(+)
*CsFAD6-2*	LOC104730201	12	4899,503–4902,312(+)
*CsFAD6-3*	LOC104727652	11	5222,054–5224,514(+)
*CsFAD7-1*	LOC104765502	1	4484,295–4487,748(+)
*CsFAD7-2*	LOC104745318	15	4751,636–4754,256(+)
*CsFAD7-3*	LOC104764868	19	5028,541–5030,994(+)
*CsFAD8-1*	LOC104734563	13	2144,948–2147,363(+)
*CsFAD8-2*	LOC104708554	8	25336,842–25339,238(+)
*CsSLD-1*	LOC104699734	7	1101,302–1103,202(-)
*CsSLD-2*	LOC104788453	5	33807,754–33809,568(+)
*CsSLD-3*	LOC104783195	4	29182,382–29184,175(+)
*CsSLD-4*	LOC104784696	5	962,282–963,994(+)
*CsSLD-5*	LOC104793590	6	25451,740–25453,806(+)

## Data Availability

Not applicable.

## Supplementary Materials

The supporting information can be downloaded at: https://www.mdpi.com/article/10.3390/ijms232314550/s1. Reference [33] is cited in the supplementary materials.
