# Supplementary figures and images for: Genome-Wide Identification and Expression Analysis of Fatty Acid Desaturase (FAD) Genes in Camelina sativa (L.) Crantz
Source: Int J Mol Sci. 2022 Nov 22;23(23):14550. doi: 10.3390/ijms232314550 (PMC9738755; doi:10.3390/ijms232314550)

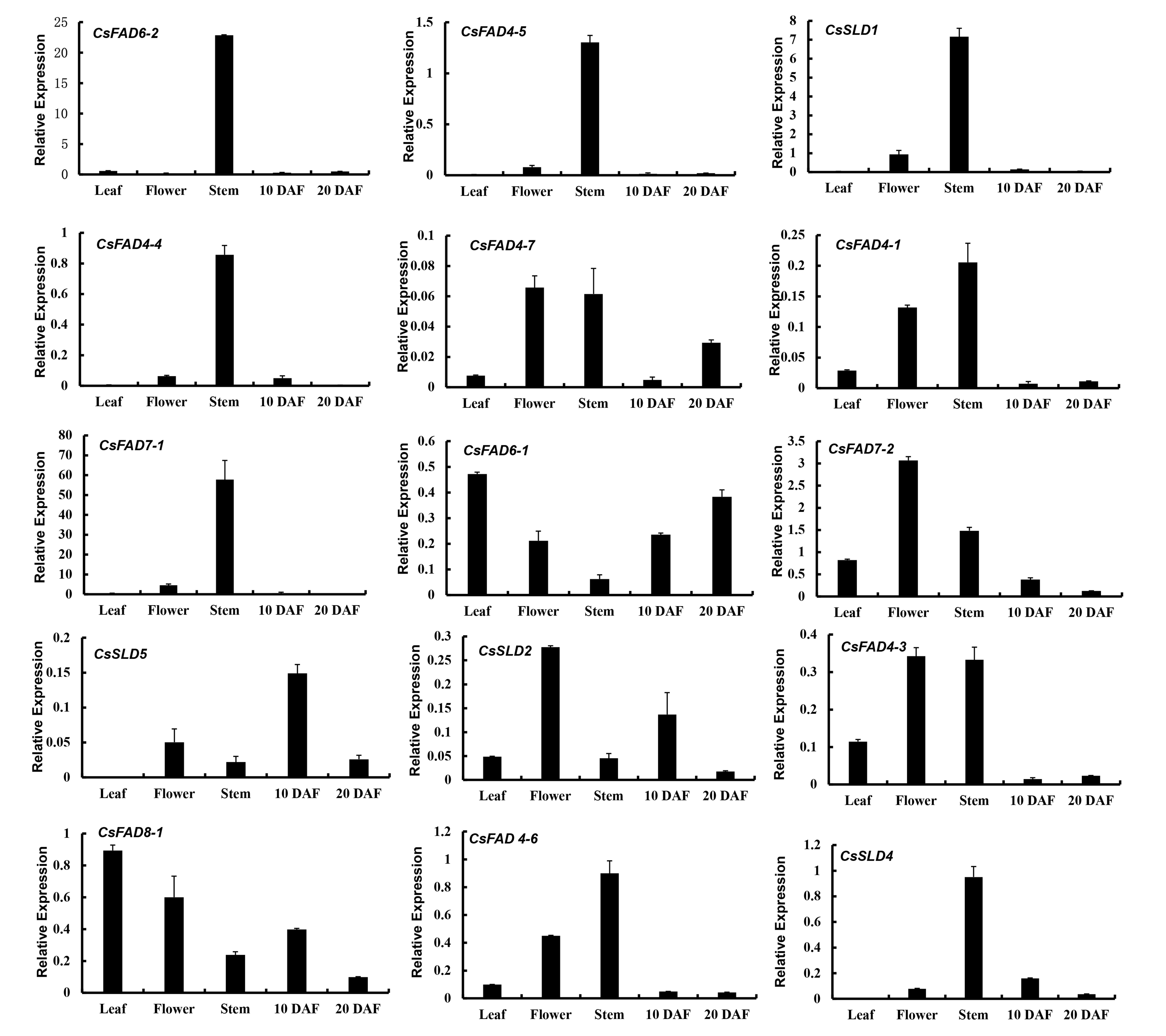

Supplement: Supplementary file 1 [file ijms-23-14550-s001.zip › Figure S1.png]

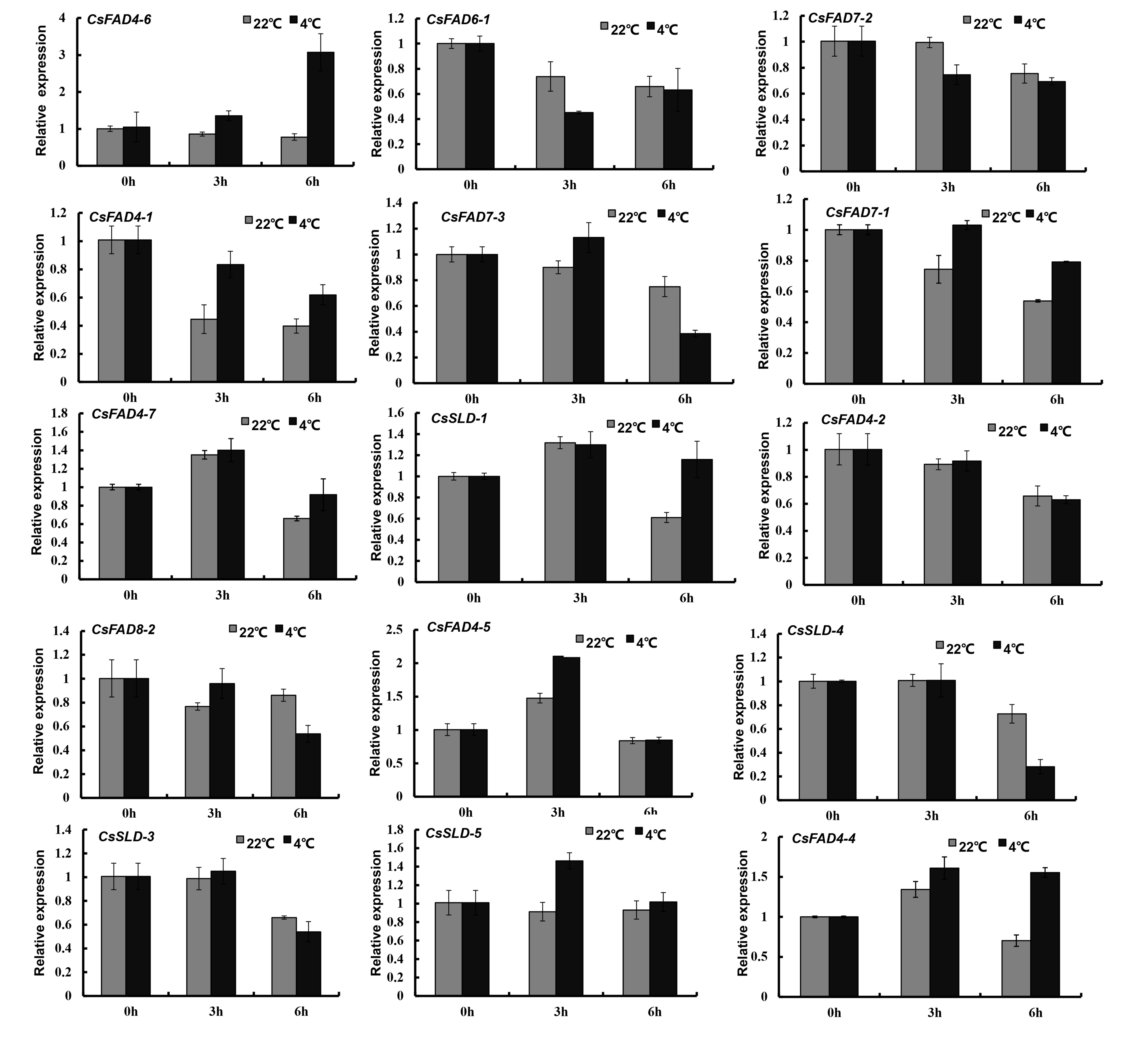

Supplement: Supplementary file 1 [file ijms-23-14550-s001.zip › Figure S2.png]

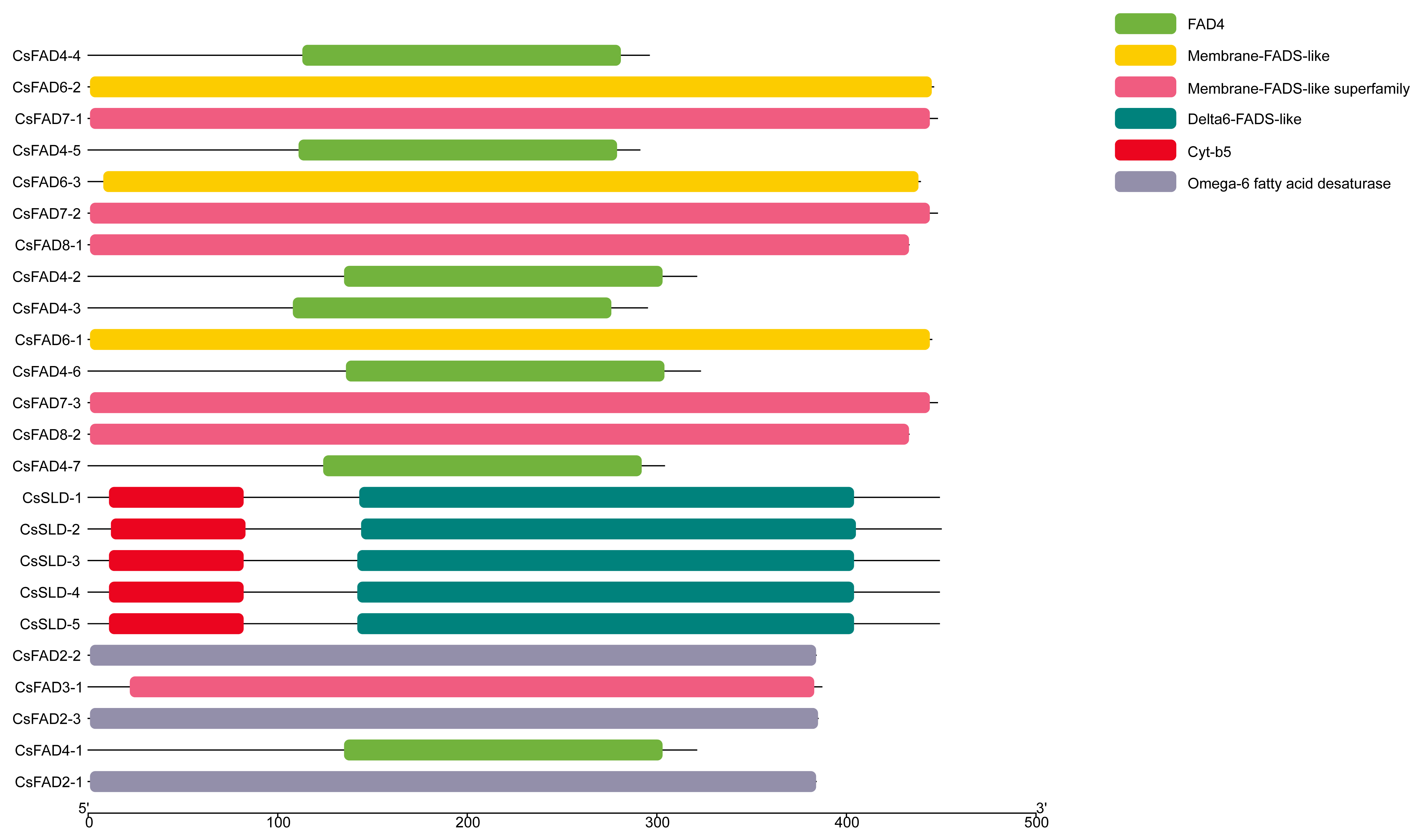

Supplement: Supplementary file 1 [file ijms-23-14550-s001.zip › Figure S3.png]

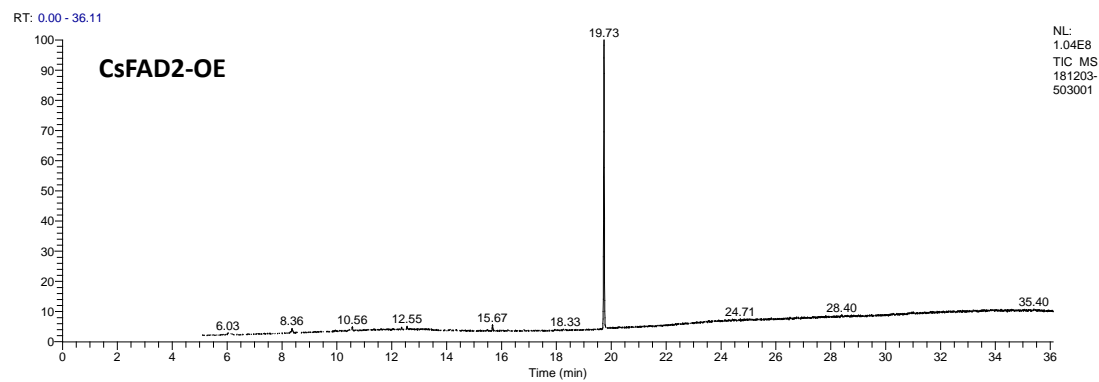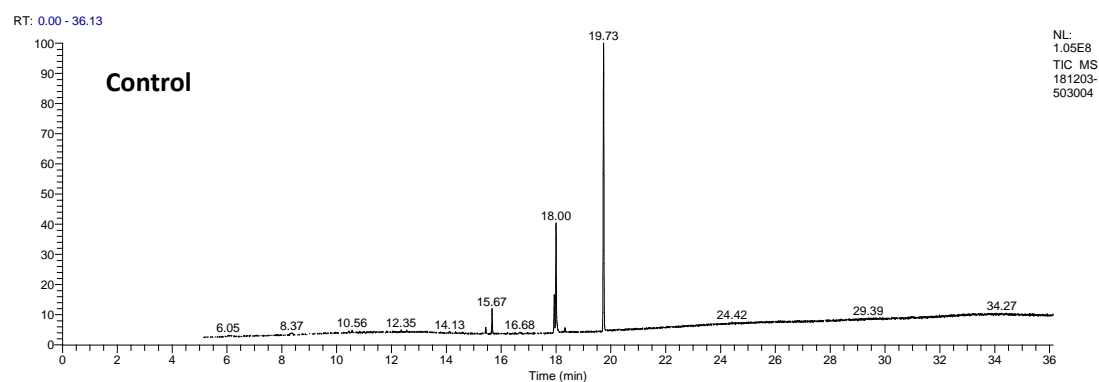

Figure S4. Chromatogram of fatty acid content determination

Supplement: Supplementary file 1 [file ijms-23-14550-s001.zip › Figure S4.pdf]

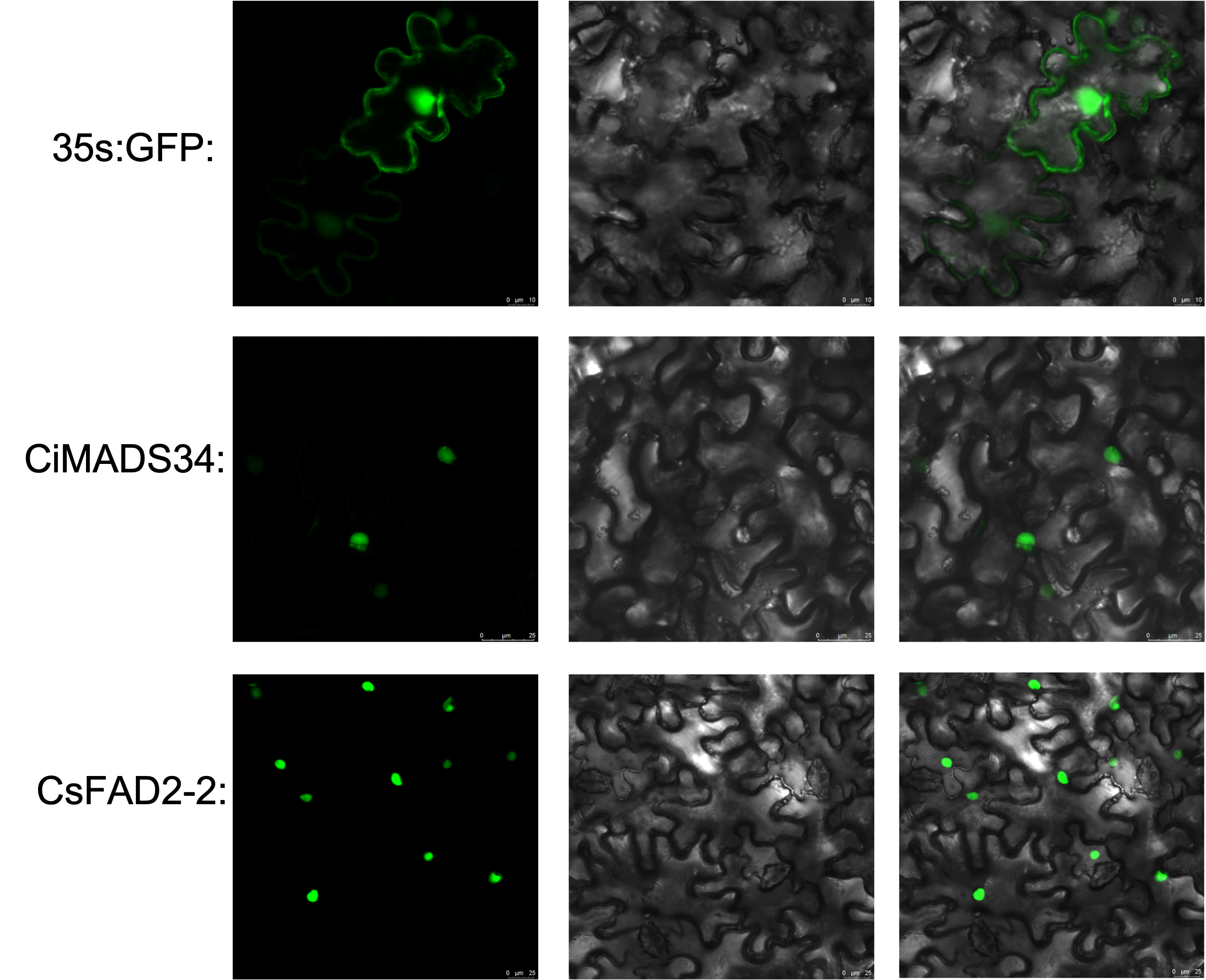

Supplement: Supplementary file 1 [file ijms-23-14550-s001.zip › Figure S5.png]
